# Are adjustable valves effective in all ages of patient? Data from the UK Shunt Registry

**DOI:** 10.1186/1743-8454-7-S1-S40

**Published:** 2010-12-15

**Authors:** Hugh Richards, Helen Seeley, John Pickard

**Affiliations:** 1 UK Shunt Registry, Academic Neurosurgery, Box 167 Addenbrooke’s Hospital, Cambridge CB2 0QQ, UK

## Background

Adjustable CSF valves have been developed by several manufacturers. These valves are more expensive, but have an advantage in that the operating pressure of the valve can be altered by the use of an external magnet. We have used data collected by the UK Shunt Registry to assess the effectiveness of these valves in reducing valve replacement for under and over-drainage using a case-control design.

## Materials and methods

The UK Shunt Registry contains data on over 55,000 CSF shunt-related procedures. Our data suggests that primary factors involved in shunt revision are patient age, diagnosis and the number of revisions a patient has had. Procedures were identified where an adjustable valve was used in a ventriculoperitoneal shunt. Of these 3,682 had an accurate diagnosis and age entered and we were able to determine the exact number of shunt revisions. A database search was performed for procedures matched for these risk factors but using a fixed-pressure valve. Matches were found for 3,262 procedures. The was dataset was further divided by the age of patient. There were 966 matched pairs aged 70 and over, 1569 matched pairs aged 17 to 69 and 727 matched pairs aged 16 and under The cumulative valve revison rate (CVRR) for up to 5 years was calculated. Only revisions where the valve was replaced for a given reason of under- or over-drainage were considered. A Logrank test was used for comparison of CVRR between adjustable and fixed-pressure valves.

## Results

In the over 70 age group, adjustable valves performed significantly better (P<0.05) from week 16 up to the 5 years of calculation. In the 17-69 age groups there was a significant lower CVRR from week 7 up to 5 years. However in the younger age group (0 to 16 years) there was no significant improvement in CVRR with an adjustable valve.

## Conclusions

Our data suggest that adjustable valves may be effective in overcoming problems due to incorrect pressure selection in adult patients. However, although appears to be no significant advantage in using an adjustable valve in the majority of paediatric patients.

